# The nucleotide composition of microbial genomes indicates differential patterns of selection on core and accessory genomes

**DOI:** 10.1186/s12864-017-3543-7

**Published:** 2017-02-10

**Authors:** Jon Bohlin, Vegard Eldholm, John H. O. Pettersson, Ola Brynildsrud, Lars Snipen

**Affiliations:** 1 Infectious Disease Control and Environmental Health, Norwegian Institute of Public Health, Lovisenberggata 8, P.O. Box 4404, 0403 Oslo, Norway; 2 Department of Chemistry, Biotechnology and Food Sciences, Norwegian University of Life Sciences, 1430 Ås, Norway

## Abstract

**Background:**

The core genome consists of genes shared by the vast majority of a species and is therefore assumed to have been subjected to substantially stronger purifying selection than the more mobile elements of the genome, also known as the accessory genome. Here we examine intragenic base composition differences in core genomes and corresponding accessory genomes in 36 species, represented by the genomes of 731 bacterial strains, to assess the impact of selective forces on base composition in microbes. We also explore, in turn, how these results compare with findings for whole genome intragenic regions.

**Results:**

We found that GC content in coding regions is significantly higher in core genomes than accessory genomes and whole genomes. Likewise, GC content variation within coding regions was significantly lower in core genomes than in accessory genomes and whole genomes. Relative entropy in coding regions, measured as the difference between observed and expected trinucleotide frequencies estimated from mononucleotide frequencies, was significantly higher in the core genomes than in accessory and whole genomes. Relative entropy was positively associated with coding region GC content within the accessory genomes, but not within the corresponding coding regions of core or whole genomes.

**Conclusion:**

The higher intragenic GC content and relative entropy, as well as the lower GC content variation, observed in the core genomes is most likely associated with selective constraints. It is unclear whether the positive association between GC content and relative entropy in the more mobile accessory genomes constitutes signatures of selection or selective neutral processes.

**Electronic supplementary material:**

The online version of this article (doi:10.1186/s12864-017-3543-7) contains supplementary material, which is available to authorized users.

## Background

Genomic nucleotide content varies greatly in bacteria, with GC content (number of same strand guanine + cytosine sites divided by DNA sequence length) ranging from less than 13% to more than 75% between individual species [1]. Variation in nucleotide composition can be substantial also within individual genomes [2]. Although the specific causes for these GC variations, both within and between species, are not known, it is predicted that a multitude of factors related to both evolutionary history and the environment are responsible [3].

Factors that show some association with genomic base composition in microbes include genome size [4–6], oxygen and nitrogen abundance [7, 8] as well as uptake of foreign DNA from conjugation, transformation and transduction [9–15]. Optimal growth temperature may influence genomic DNA composition and although this is a field of debate [16–19], there is some evidence for a role of growth temperature in shaping the GC content of individual genes [20] and ribosomal RNA [21]. Mutations are generally biased towards AT-richness mainly due to the process of deamination of cytosine [22, 23]. A strong positive correlation between fitness and GC content was found in *Escherichia coli* over-expressing synthetic versions of a GFP gene with varying GC content, suggesting that increased GC content in bacteria may be associated with increased selective pressures [24]. GC-richness may be driven by selection for more stable DNA as stacking (and breaking) of guanine and cytosine typically requires more energy than stacking of adenine and thymine [25]. GC-rich genomes may also have been subjected to selection for more energetically favorable amino acid usage, as GC-rich codons code for less energy-requiring amino acids than AT-rich codons [26]. Moreover, many bacteria “silence” foreign AT rich DNA sequences, often found in phages [27, 28]. On the other hand, relaxation of selective pressures has been suggested to drive symbiotic microbial genomes towards AT-richness due to AT mutation bias and loss of DNA repair genes [29]. Non-coding parts of microbial genomes have been found to be more AT-rich than the coding parts and this could be due to relaxed selective pressures in non-coding regions as compared to coding regions [30].

Changes in genomic nucleotide composition could also be a consequence of selectively neutral processes. Indeed, a presumably selectively neutral process known as GC-biased gene conversion (gBGC) could be widespread in bacterial genomes [31]. Another putatively selectively neutral process, termed “amelioration”, seems to even out differences in base composition between integrated DNA from foreign sources, which is often AT-rich [6], and host chromosomes [32, 33]. While there are several examples that support all the above claims, there are also findings that question their general validity. Examples include obligate intracellular microbes with GC rich genomes having undergone severe genome degradation [34] as well as a lack of findings supporting the notion that increased GC content stabilizes DNA (although increased AT content seem to be destabilizing [35]). How the presumably selectively neutral processes of amelioration and gBGC are operating on bacterial genomes is also not completely understood [36, 37]. Hence, it is evident that the fundamental selective processes shaping base composition in microbial genomes are multi-factorial and complex.

The study of pan-genomes [38] is, amongst other things, concerned with classifying genes as conserved or accessory. Typically, the conserved genes are assumed to be linked to important functions related to cell maintenance, such as metabolism, DNA housekeeping and repair and therefore termed core genes. Accessory genes, on the other hand, may increase fitness due to a particular environmental niche or short-term exposure such as antibiotic challenge [39]. It is presumed that core genomes are subjected to stronger purifying selection than the accessory genome, since they have been retained in all strains of a species [5, 38, 40–44]. Hence, analyzing the intragenic nucleotide composition in microbial core and accessory genomes could reveal how selective pressures, as well as putative selectively neutral processes such as gBGC and amelioration, affect base composition. By examining the intragenic base composition of core and accessory genomes comprising 731 prokaryotic strains from 36 different species, 28 genera and 10 phyla, for which closed genomes of > 10 strains were available, we explored whether differences could be detected between the mentioned genomic regions. These results were in turn compared with corresponding genome-wide analyses. We restrict this analysis to coding regions, i.e. non-coding regions were excluded, since it is less clear if non-coding regions would be subject to similar selective pressures as coding regions.

## Results

### GC content in core, accessory and whole genomes

**Fig. 1:**
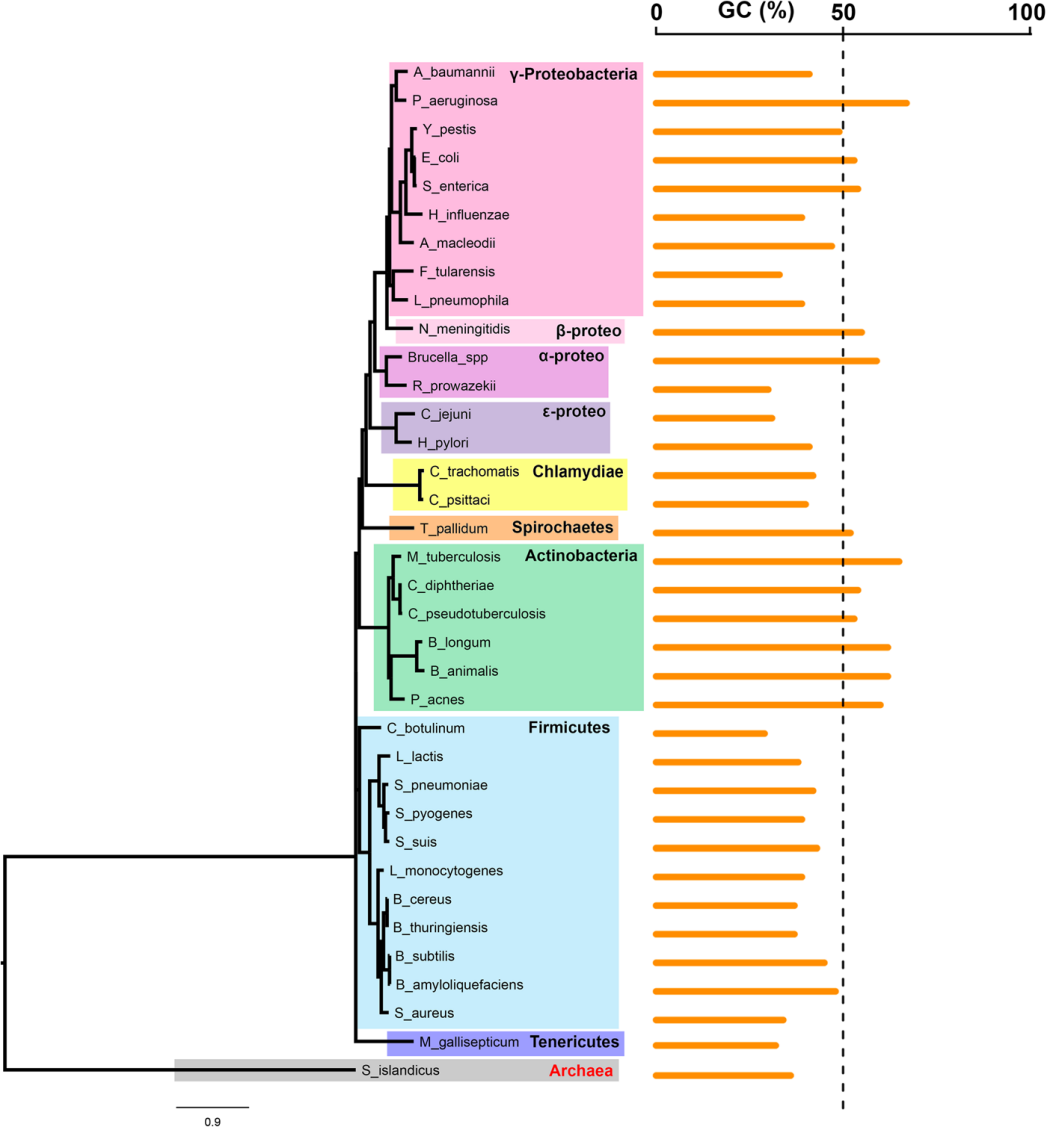
%GC and phyla. The 16S based phylogenetic tree demonstrates how % GC varies at the phylum, genus and species levels for all microbes included in the study

**Fig. 2:**
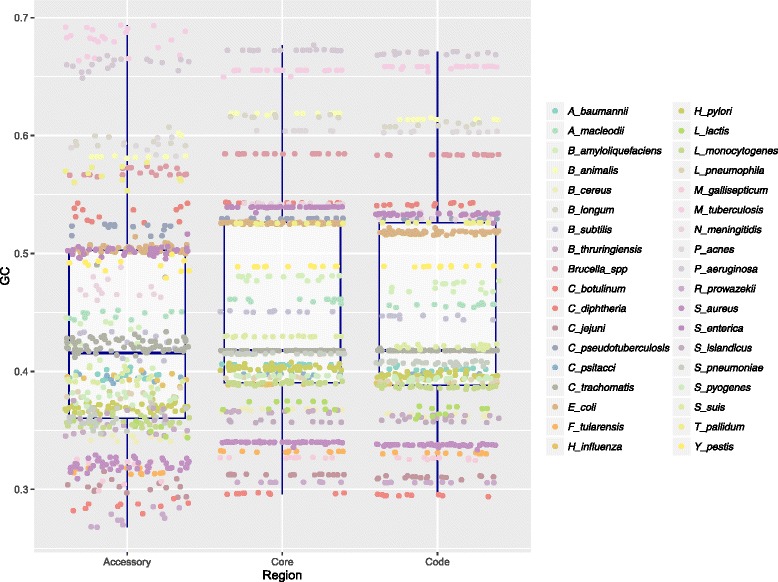
Genomic GC content. The box plot indicates how GC content (*vertical axis*) is distributed in the core, accessory and whole genome regions (*horizontal axis*) of all strains analyzed in the present study, colored according to species as indicated by the legend to the right

**Fig. 3:**
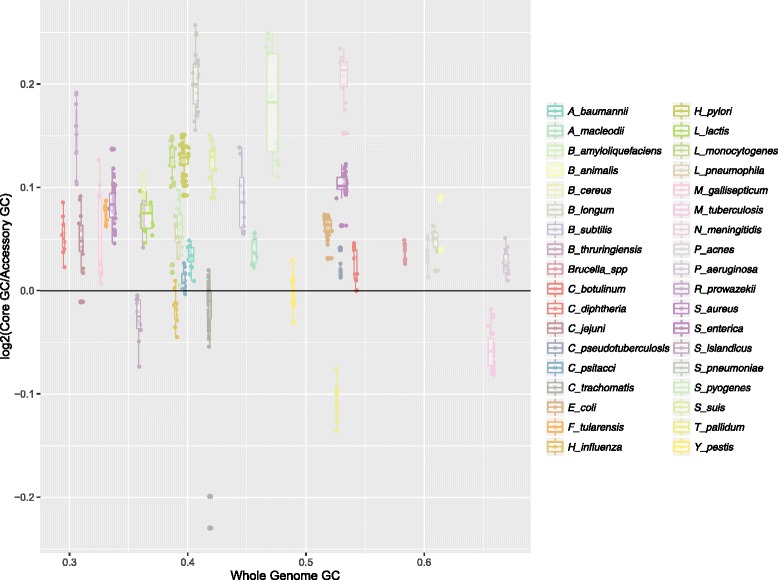
Differences between core genome and accessory genome %GC. The figure shows log_2_-transformed GC content differences (*vertical axis*) between core and accessory genomes for all species considered in the present study (legends to the right), with respect to their corresponding genome-wide GC content (*vertical axis*)

To examine differences in nucleotide composition between the coding regions of the core genome, accessory genome and the whole genome (i.e. all genes, including accessory and core genomes) of the 36 species, (Table 1) we fitted a linear mixed-effects model with GC content as the response variable and sequence type (i.e. core, accessory and whole genome) as the explanatory variable (See Additional file 1 for more information regarding the statistical models). The taxonomic ranks of phylum, genus, and species were added as random effects. However, adding phylum as taxonomic level (See Fig. 1) did not result in improved models (*p = 0.14*, maximum likelihood ratio test) and no association was found between phylum and %GC (*p = 0.625*, ANOVA) using a phylogenetic regression model adjusting for Brownian motion correlation structure between the branches (See Methods for more details). Including both genus and species as hierarchical random effects resulted in significantly improved models as compared to species only (*p = 0.008*, maximum likelihood ratio test) therefore all mixed-effects models will henceforth include the two levels genus and species as random effects, but not phylum. The regression model with %GC as the response and intragenic region (i.e. core, accessory or whole genome) as the explanatory variable indicated that GC content was significantly higher (See Figs. 2 and 3 as well as Additional file 2), on average, in the core part of the genome (*p < 0.001*) than the whole (*p < 0.001*), and accessory genomes (*p < 0.001*). The GC content in the accessory part of the genomes was significantly lower than in whole genomes (*p < 0.001*).

**Table 1:** Core genome characteristics

Species	Strains #	Size in mb (full)	Size in mb (accessory)	%GC (code)	%GC (accessory)	%GC (core)
*A_baumannii*	16	3,9	1,04	40,19	39,67	40,59
*A_macleodii*	13	4,5	1,03	45,66	44,8	46,05
*B_amyloliquefaciens*	16	4,01	0,45	47,28	42,39	47,98
*B_animalis*	12	1,94	0,23	61,36	58,53	61,88
*B_cereus*	13	5,27	0,81	36,25	34,39	36,77
*B_longum*	10	2,46	0,61	60,84	59,57	61,61
*B_subtilis*	12	4,1	0,46	44,71	42,36	45,07
*B_thruringiensis*	12	5,51	0,88	36,23	34,91	36,59
*Brucella_spp*	20	3,3	0,16	58,36	56,9	58,45
*C_botulinum*	10	3,95	0,43	29,48	28,6	29,66
*C_diphtheria*	13	2,47	0,31	54,15	53,42	54,3
*C_jejuni*	16	1,67	0,27	31,05	30,22	31,25
*C_pseudotuberculosis*	15	2,32	0,11	52,93	52,16	52,97
*C_psitacci*	10	1,16	0,04	39,76	39,47	39,74
*C_trachomatis*	78	1,04	0,01	41,78	42,34	41,77
*E_coli*	62	5,01	1,51	51,79	50,35	52,58
*F_tularensis*	12	1,9	0,14	33,08	31,53	33,21
*H_influenza*	10	1,9	0,3	38,97	39,4	38,92
*H_pylori*	53	1,62	0,29	39,64	36,93	40,31
*L_lactis*	11	2,45	0,53	36,86	35,32	37,14
*L_monocytogenes*	30	2,93	0,3	38,66	38,84	39,01
*L_pneumophila*	12	3,33	0,7	39,12	38,03	39,48
*M_gallisepticum*	12	0,97	0,05	32,65	31,64	32,69
*M_tuberculosis*	23	4,4	0,36	65,86	68,13	65,49
*N_meningitidis*	14	2,22	0,26	53,09	47	54,2
*P_acnes*	10	2,51	0,17	60,30	59,06	60,4
*P_aeruginosa*	18	6,43	0,89	66,96	66,03	67,26
*R_prowazekii*	10	1,11	0,01	30,58	27,57	30,61
*S_aureus*	49	2,82	0,28	33,78	32,08	33,99
*S_enterica*	42	4,78	0,64	53,34	50,2	53,92
*S_islandicus*	10	2,65	0,37	35,76	36,43	35,74
*S_pneumoniae*	27	2,11	0,26	40,73	36,07	41,5
*S_pyogenes*	19	1,85	0,23	39,38	37,95	39,63
*S_suis*	18	2,09	0,4	42,11	39,42	42,97
*T_pallidum*	11	1,14	0,01	52,60	56,63	52,55
*Y_pestis*	12	4,58	0,28	48,92	49	48,9

### Base composition and bias

**Fig. 4:**
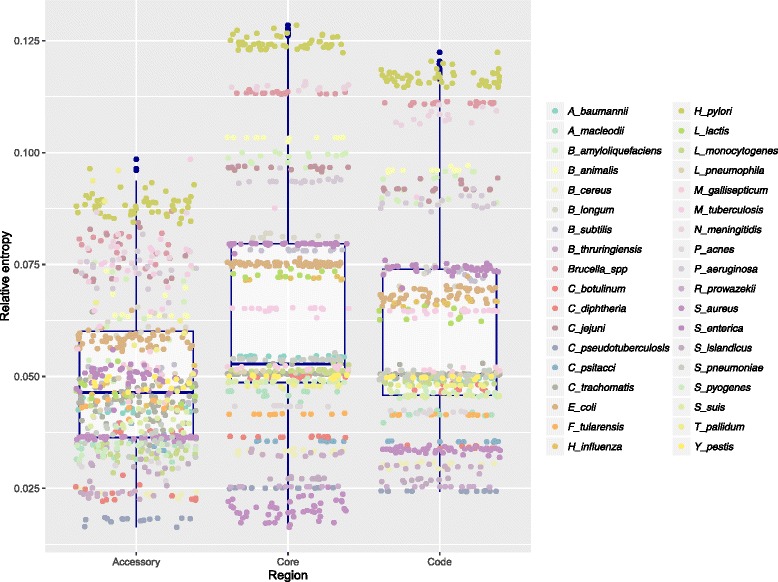
Relative entropy. The box-plot demonstrates how relative entropy (*horizontal axis*) is distributed genome-wide and among accessory and core genomes (*horizontal axis*). All strains are colored according to the species they belong to, as designated by the legend to the right

To further explore whether the base composition of core and corresponding accessory genomes were subjected to different selective pressures we used the concept of relative entropy [9]. This measure indicates whether genomic oligonucleotide patterns, such as codons, are observed more or less often than expected from genomic mononucleotide frequencies (i.e. AT/GC content). High relative entropy indicates a great distance between observed- and expected oligonucleotide frequencies, suggesting that the oligonucleotide frequencies are biased, most likely due to selection or putative selective neutral forces [31, 32]. Loosely speaking, low relative entropy points to more randomly distributed oligonucleotide frequencies, something that would be expected in a DNA sequence that has undergone genetic drift [9]. Examining differences in relative entropy between intragenic core, accessory and whole genomes, using an identical mixed-effects regression model to the one based on GC content discussed above, but with relative entropy as the response rather than GC content, we found significantly higher relative entropy in the core part of the genome as compared to the whole- (*p < 0.001*) and accessory genomes (*p < 0.001*). Genome-wide relative entropy was significantly higher than in the accessory part of the genomes (*p < 0.001*) (Fig. 4 and Additional file 3).

### GC content variation differences between genetic regions

**Fig. 5:**
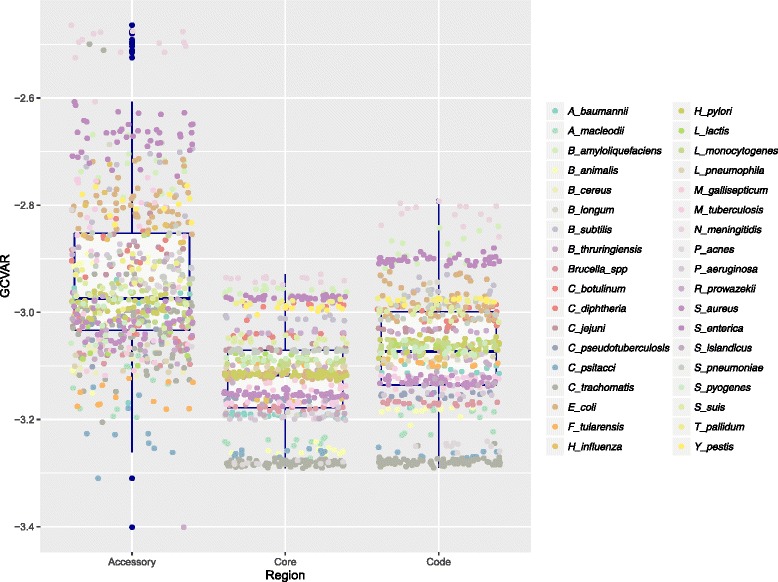
GCVAR. The figure shows a box-plot of how GCVAR (*vertical axis*) varies genome-wide and in the core and accessory genomes (*horizontal axis*) considered in the present study. All strains are colored according to the species specified by the legend to the right

Changing the response variable of the mixed-effects regression model described above to within-genome GC content variation, referred to as GCVAR [2], we found that the core genome exhibited significantly lower GCVAR than the corresponding accessory (*p < 0.001*) and whole genomes (*p < 0.001*). Genome-wide GCVAR was, in turn, significantly lower than accessory genome GCVAR (*p < 0.001*). This indicates that, on average, GC content varies significantly less within the core parts of the coding genome than in the rest (Fig. 5 and Additional file 4). Lower GCVAR has also previously been associated with increased selective constraints [37] as a lower variation in GC content may be an indication of purifying selection acting on base composition. Genome-wide GCVAR was significantly lower than for accessory genomes (*p < 0.001*) [29].

### Oligonucleotide- and GC content bias in core, accessory and whole coding genomes

It has been shown that genome homogeneity in prokaryotes, as measured using oligonucleotide frequencies, is associated with genomic %GC [45]. In other words, the more GC rich the genome the more similar the oligonucleotide usage appears to be. As has been previously observed [9], we find a relatively weak correlation, using mixed-effects linear regression models with taxonomy as the random effect, between GC content and relative entropy on the accessory part of the genomes (*p = 0.005*), but not on the corresponding core (*p = 0.19*) or whole genome regions (*p = 0.45*). A positive correlation between relative entropy and GC content in the accessory part of the genomes (See Additional file 5) may support gBGC and/or amelioration as the accessory genome is presumably more mobile than the core genome implying that the accessory genes have, on average, been subjected to considerably more frequent recombination events [12]. In this regard, it is interesting to note that an association between GC content and codon bias is predicted to result from gBGC [31].

### Exceptions to the observed trends

The majority of species discussed here did adhere to the tendency that GC content and relative entropy was higher, and GCVAR lower, in the core genomes, compared to the corresponding accessory genomes and whole genomes. There were however some species were such differences were negligible or even reversed in one or all the measures considered. These species were typically pathogens associated with an intracellular lifestyle like *Rickettsia prowazekii*, *Mycobacterium tuberculosis*, *Chlamydia spp., Treponema pallidum, Mycoplasma gallisepticum and Francisella tularensis* [29]. In addition to the intracellular pathogens the free-living pathogens *Haemophilus influenzae*, *Clostridium botulinum* as well as the extremophile archaeon *Sulfolobus islandicus* exhibited some deviance from the common trend of higher core genome GC content and relative entropy in addition to lower GCVAR. These strains possessed large core genomes with a median fraction of 97% of the genome being classified as core versus 83% of the other species (*p < 0.001*, Wilcoxon rank sum test). Core genome GC content was higher in 608 out of 731 strains, core genome relative entropy was higher in 721 strains and GCVAR was lower in the core genomes of 677 strains, all compared with the respective measures applied on the corresponding genome-wide regions (See Additional file 6 for more information).

## Discussion

### Influence of selective pressures on base composition

As mutations in bacteria are AT-biased [22] it has not been obvious how GC rich microbes can exist. The retention of more energetically expensive and nitrogen-heavy guanine and cytosine nucleotides across core genomes suggests that selective pressures are at work, but identifying and classifying these processes is challenging. Our findings seem to indicate that core genome GC content is conserved by purifying selection, as microbial core genomes must over time have been subjected to stronger purifying selection than the rest of the genome and certainly the more mobile genes of the accessory genomes [41, 44]. Contrarily, in recently emerged clonal strains, traces of purifying selection are in fact more dominant in the accessory part of the genomes due to the transfer of mobile genes from other organisms that have already purged them of fitness-decreasing *de novo* mutations [12]. Thus, in such clonal strains, purifying selection has not had the opportunity to remove recently emerged *de novo* mutations from the core genome, quite the opposite of what is observed between phylogenetically more diverse strains [12]. The strains included in the present study are predominantly inter-clonal and therefore it is presumed that purifying selection dominates the core genomes and that the accessory genomes are marked by recombination events and having been subjected to vastly different selective pressures than the corresponding core genomes [12, 36]. Our results seem to indicate that this is expressed as greater variance in %GC, lower relative entropy, and higher GCVAR in the accessory genomes of each species’ strains, as compared to the corresponding core genomes and genome-wide, where the variation between strains is remarkably similar, as can be observed in Figs. 2, 3, 4 and 5 (and Additional files 2, 3, 4 and 5).

Since mutations in bacteria are AT-biased, the purging of deleterious mutations in the core genome may act to conserve GC content as compared to the rest of the genome [42]. Moreover, as the accessory genome may be subjected to weaker selective forces than the core genome, one might assume that fitness decreasing mutations are better tolerated in the non-core parts of the genome [42]. Two recent studies [8, 26] may also provide important pieces to the puzzle of how microbial genomes can maintain GC-richness. Chen et al. demonstrated that AT rich codons are translated into more energy requiring amino acids than GC rich codons. Thus, there appears to be a selective trade-off between energy requiring amino acids and nucleotides, respectively, so that genomic GC richness is maintained or, in some circumstances, even increased. Seward and Kelly provided further evidence that increased environmental nitrogen abundance can affect base composition in the direction of higher GC content [8, 46].

### The influence of selectively neutral processes on base composition

Apart from selection, selectively neutral processes may also be involved in shaping genomic GC content. One such process, namely gBGC, has been observed in mammals [47] and appears to be widespread in eukaryotes [48]. A recent study now provides evidence of gBGC in bacteria and archaea [31]. Another, putative selective neutral process, referred to as amelioration, was described by Lawrence and Ochman in 1997 [32]. This process could be at work in many prokaryotes having taken up DNA from phylogenetically distant sources. The concept of amelioration, in short, asserts that foreign DNA integrated into a host chromosome, having a substantially different base composition, will eventually attain a progressively more similar base composition to that of the host chromosome. The exact details regarding this process are not completely understood, but the process of amelioration has been noted in several instances [3, 9, 49–53].

Foreign DNA sequences, like phages and plasmids, are often more AT rich than the host chromosome [6, 27]. If the base composition of integrated foreign DNA is becoming progressively more similar to that of the host chromosome, as is hypothesized by the process of amelioration, this would in many instances imply that the foreign DNA is becoming gradually more GC rich. Since gBGC is assumed to increase GC content in recombined DNA it could, in principle, mean that the process of gBGC is related to amelioration (or vice versa). As the effects attributed to these processes are presumed to be weak, they might also be confounded by selection [1, 32, 36, 37]. Indeed, the positive correlation we observed between %GC and relative entropy in the accessory genomes appears to advocate selective neutral processes, such as gBGC or amelioration. However, we would expect the impact of such processes to decrease in influence with progressively more GC-rich genomes, but this is not supported by our findings, which are largely linear, indicating no change (See Additional file 5).

### Environment and phylogeny

In summary, our statistical models suggest that genomic base composition in prokaryotes is strongly affected by a phylogenetic “inertia” at the species level, less so at the genus level and significantly not at the phylum level and above (Fig. 1). Population size may mediate selective pressures through this phylogenetic “inertia” in the sense of genome streamlining [54] due to high population density, through Muller’s ratchet [55] if the population density is low, or through other capacities set by the environment [36]. Selection for energetically expensive nucleotides and/or amino acids is, on the other hand, predominantly driven by the environment, affecting both positive and negative selection. Phylogeny and environment will thus both contribute to the effect that recombination has on microbial populations, which in turn will have a spiraling impact on genomic base composition. Following this line of reasoning, the increased %GC we find in the majority of prokaryotic core genomes seems to be maintained by phylogenetic inertia while the more varied and AT rich base composition in the corresponding accessory and non-core parts of the genome may be more influenced by the environment and the base composition of other hosts. Indeed, the species with core genome %GC and relative entropy similar to or lower, and GCVAR higher, than the non-core genome were mostly intracellular suggesting that recombination and genetic exchange with other microbes is less frequent than that of the other species [29], something that was also apparent by the significantly larger core genomes in these species. Deleterious *de novo* mutations and horizontally acquired defective genes are purged through purifying selection over time, the degree to which may be, amongst other factors, determined by effective population size, which is small for intracellular microbes [29, 56]. As both uptake of phages and mutations are AT-biased, removal or purging of such genetic regions will thus, in most instances, retain genomic %GC. So will homologous recombination, and it is these processes we believe dominate the differences in base composition we observe between core and corresponding accessory genomes. Our results cannot conclude whether neutral selective processes, such as gBGC and/or amelioration, or selection are more pronounced in the accessory genomes. While the strength of both positive and negative selection will vary between species and environments, the effects of selective neutral processes should remain, more or less, constant between environments but vary between species [36]. Hence, the relative strengths of selective and neutral processes on prokaryotic species depend on both phylogenetic and environmental factors and will hopefully be illuminated further in the time to come.

## Conclusions

We find that the coding regions in core genomes are significantly more GC-rich, has less GC content variation and higher relative entropy (i.e. more biased oligonucleotide distributions) than the coding regions in the rest of the corresponding genomes. Exceptions to these findings were mostly detected in intracellular bacteria. Due to the fact that core genes are present in almost all strains, and therefore subjected to higher levels of purifying selection than the rest of the corresponding genomes, our results indicates that there is an association between base composition and selective pressures. More specifically, purifying selection seems to be associated with increased GC content.

## Methods

For our results to be as reliable as possible, with regard to statistical testing, only species having 10 or more strains with fully sequenced and closed genomes were included into the study. This resulted in a total 731 closed genomes, and corresponding coding sequences (both gene- and protein sequences), comprising 36 species from 28 genera and 10 phylogenetic groups all of which were downloaded from NCBI January 7 2016 [57]. Information regarding all species and strains used in the present study can be found in Additional file 6 and Table 1.

The pan-genome analysis targeted each species separately, except for *Brucella*, where the analysis was performed for the entire genus. Coding genes were translated into proteins, and compared all-against-all using BLAST v.2.4.0 [58] and the “micropan” R-package [59]. A vignette is available in the “micropan” package for exact details on how to perform the analysis. Sequences were clustered into gene families using a complete linkage clustering with a threshold BLAST-distance of 0.75 [59]. A BLAST-distance between two coding genes *A* and *B* is

$$ d\left( A, B\right)=1-\frac{1}{2}\left(\frac{b\left( A; B\right)}{b\left( A; A\right)}+\frac{b\left( B; A\right)}{b\left( B; B\right)}\right) $$

Where *b*(*A*; *B*) is the BLAST-score for the alignment of gene *A* and *B*, with *A* as query, i.e. *b*(*A*; *A*) is the self-alignment producing maximum score (exact identity). ‘Complete linkage gene family’ means that a gene belongs to a gene family if its BLAST-distance to all other members in the family is below 0.75.

For each pan-genome we excluded all singleton genes (genes found in 1 strain only) since these are expected to contain a significant proportion of mis-annotations from the gene prediction. Core genes were defined as those present in at least 95% of the strains within the pan-genome. The accessory genome then contains all other gene families, i.e. those present in at least two strains but less than 95% of the strains.

The 16S phylogeny was created based on alignments of 16S genes extracted from one strain from each species using MAFFT v7.123b [60]. The 16S gene alignments are available in Additional file 7. RAxML v8.2.4 [61] was subsequently employed to create a phylogenetic tree that was bootstrapped 500 times. To examine the phylogenetic differences in genome-wide %GC at the phylum level, a generalized least squares model (GLS) was fitted with %GC as the response- and phylum as the explanatory variable. The 16S based phylogenetic tree was added to the GLS model to adjust for phylogenetic structure which was found to be most appropriately modeled as a Brownian motion using Pagel’s λ [62] (*p < 0.001*, maximum likelihood ratio test). This analysis was performed using the R-packages “APE” and “nlme” [63, 64].

Relative entropy was based on the Kullback–Leibler divergence, calculated as the distance between the observed overlapping frequencies of trinucleotides *f*_*XYZ*_ over expected frequencies of trinucleotides computed using the mononucleotide frequencies *f*_*X*_ *f*_*Y*_ *f*_*Z*_ of each corresponding trinucleotide [9]. Intra-genomic GC content variation, GCVAR [2], was calculated as the log-average difference of GC content using 100 bp sliding windows subtracted from the GC content of the sequence type (i.e. intragenic core and accessory genomes as well as genome-wide):

$$ GCVAR= \log \left(\frac{1}{N}{\displaystyle \sum_{i=1}^N}\left|{D}_i\right|\right),\ {D}_i= G{C}_i- G C $$

All stated mixed-effects regression analyses were carried out using the package “lme4” in R [65]:

$$ {y}_{ijk}={\beta}_0+{\beta}_1{x}_{ijk}+\boldsymbol{Z}{u}_{ijk}+{\epsilon}_{ijk} $$

The response variable *y*_*ijk*_ represents either GC content, GCVAR or relative entropy, while *β*_*0*_ is the estimated intercept parameter. The explanatory variable *x*_*ijk*_ represents either sequence type (i.e. whole genome, core and accessory genomes), or GC content, while *β*_*1*_ is the associated parameter that is computed by the regression model. The computed random effects, accounting for variance differences within phyla (*i*), genus (*j*) and species (*k*) *u*_*ijk*_ are found in the covariance matrix **Z**. The errors *ε*_*ijk*_ are assumed to be normally distributed with mean zero and variance equal to one. Parameter estimates from the mixed effects models were computed using the method described by Satterthwaite and implemented in the R-package “lmerTest” [66]. The same package was also used for the likelihood ratio test–based comparisons of the mixed-effects regression models. Multiple comparisons of the explanatory variables were performed using the Tukey Honest significance difference test from the “multcomp” package [67]. All statistical regression models were assessed by plotting the fitted model to the data as well as using qq- and distributional plots. The comparison of core genome fractions was performed using the Wilcoxon rank sum test. All figures were made using the package “ggplot2” with R [68].

## Additional files

Additional file 1: Statistical model estimations and results. (TXT 2 kb)
Additional file 2: GC content in core, accessory and whole genomes. (PDF 31 kb)
Additional file 3: Relative entropy in core, accessory and whole genomes. (PDF 31 kb)
Additional file 4: GCVAR in core, accessory and whole genomes. (PDF 31 kb)
Additional file 5: Relative entropy plotted against accessory genome %GC together with regression estimates. (PDF 69 kb)
Additional file 6: Dataset containing all data used in the statistical models together with the strains ID’s and accession numbers. (XLSX 196 kb)
Additional file 7: 16S gene alignments in FASTA format. (TXT 38 kb)
