# Supplementary figures and images for: The nucleotide composition of microbial genomes indicates differential patterns of selection on core and accessory genomes
Source: BMC Genomics. 2017 Feb 10;18:151. doi: 10.1186/s12864-017-3543-7 (PMC5303225; doi:10.1186/s12864-017-3543-7)

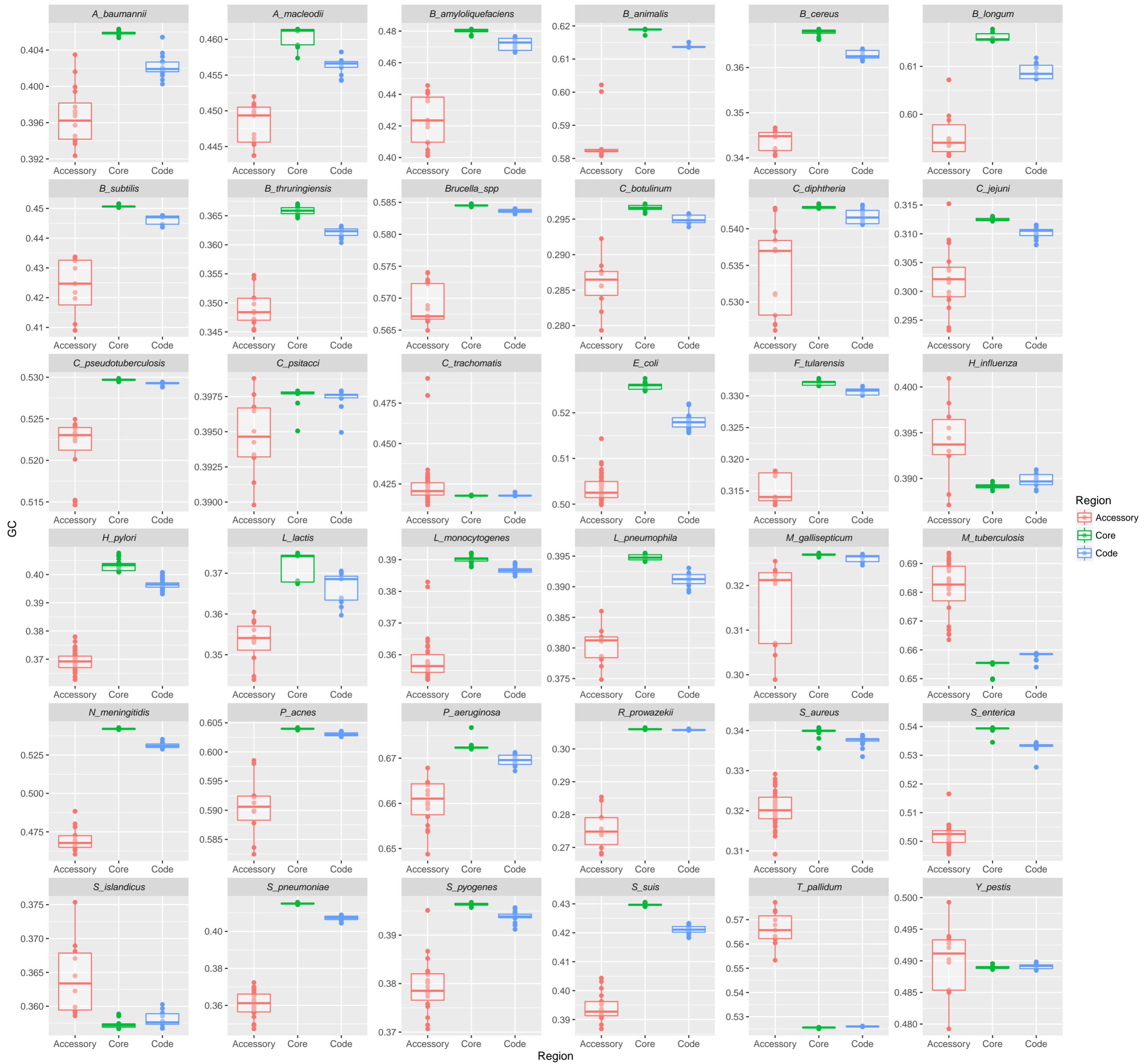

Supplement: Additional file 2: — GC content in core, accessory and whole genomes. (PDF 31 kb) [file 12864_2017_3543_MOESM2_ESM.pdf]

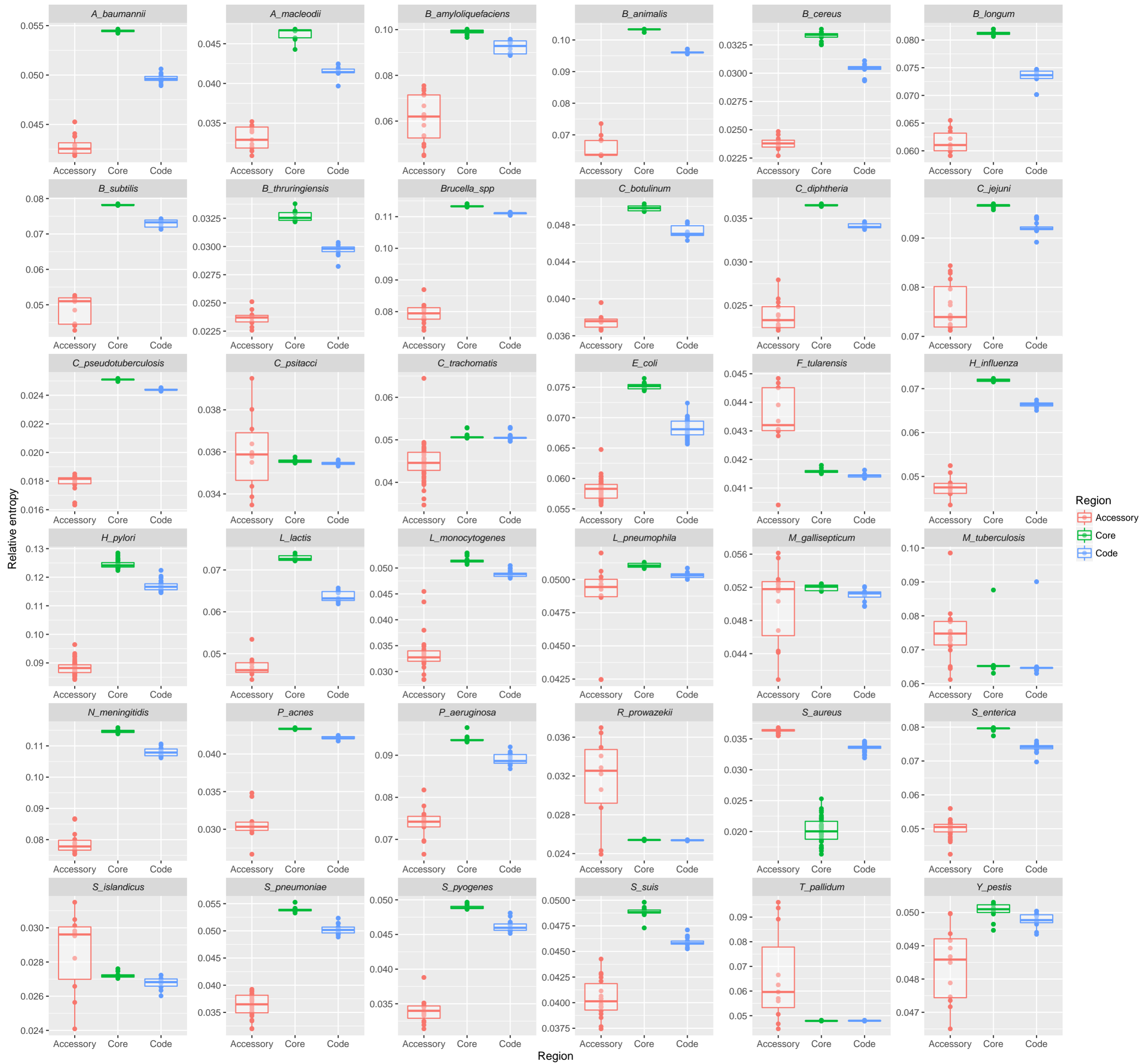

Supplement: Additional file 3: — Relative entropy in core, accessory and whole genomes. (PDF 31 kb) [file 12864_2017_3543_MOESM3_ESM.pdf]

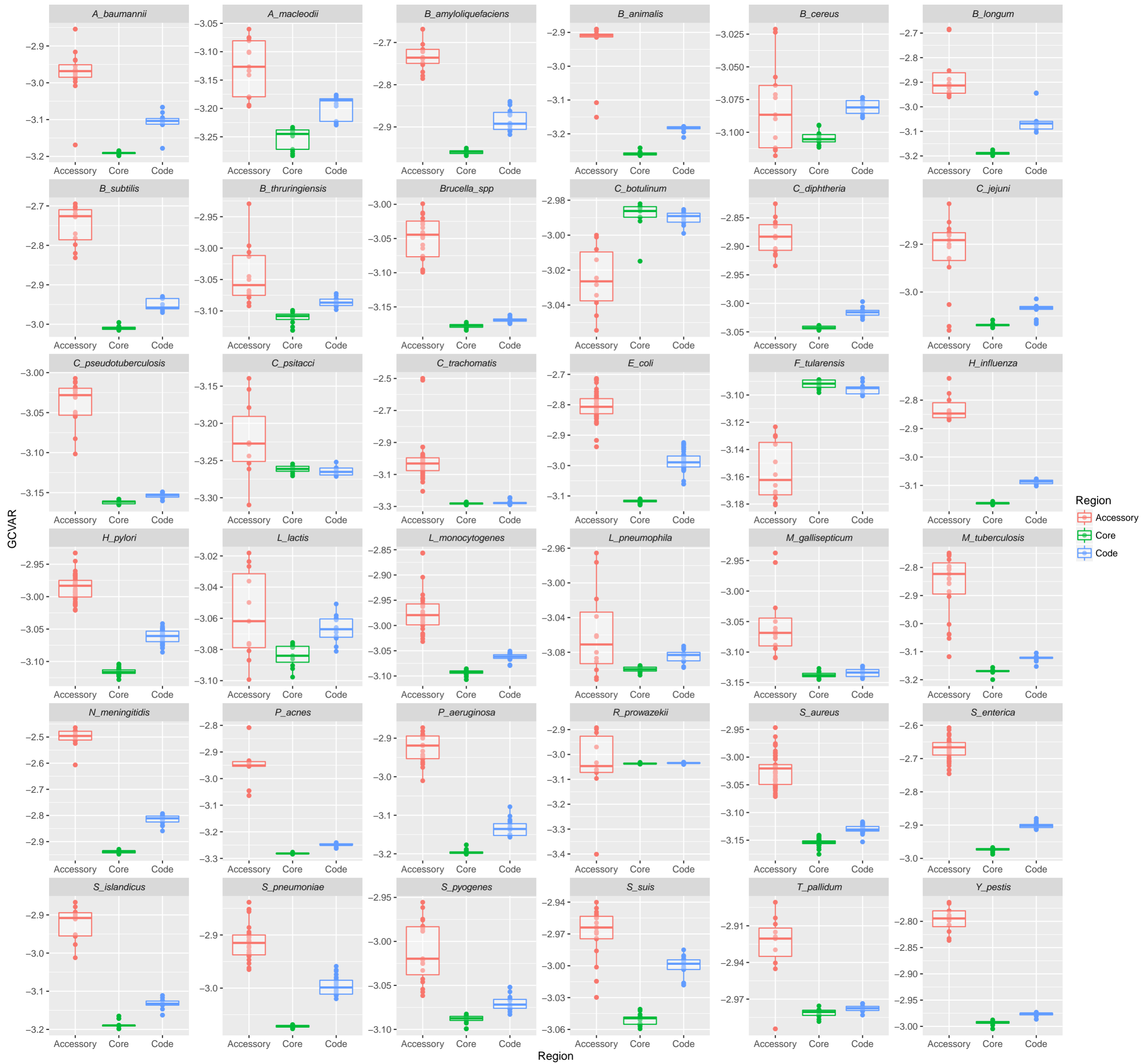

Supplement: Additional file 4: — GCVAR in core, accessory and whole genomes. (PDF 31 kb) [file 12864_2017_3543_MOESM4_ESM.pdf]

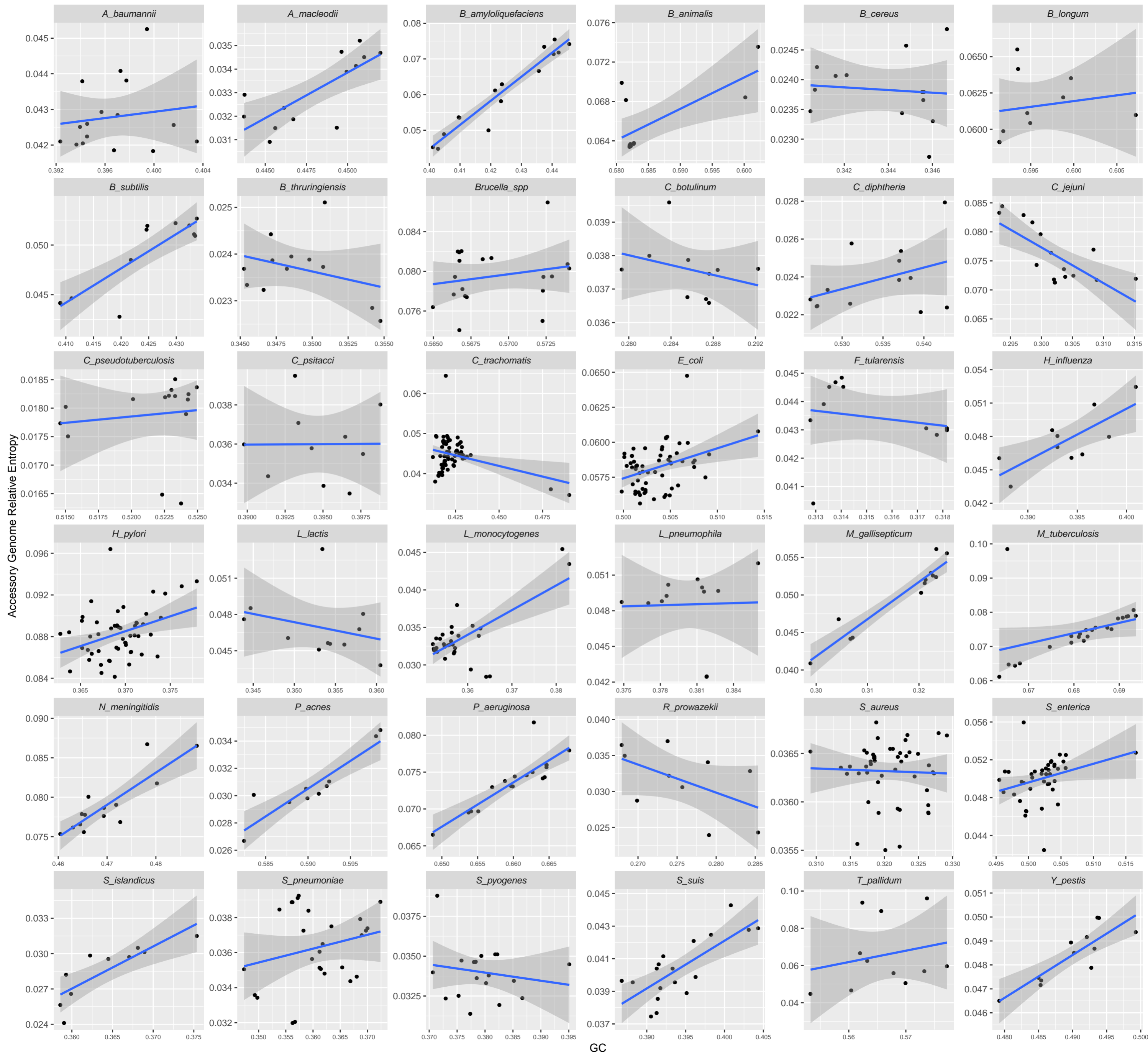

Supplement: Additional file 5: — Relative entropy plotted against accessory genome %GC together with regression estimates. (PDF 69 kb) [file 12864_2017_3543_MOESM5_ESM.pdf]
